# Tumor necrosis factor and its receptors in the neuroretina and retinal vasculature after ischemia-reperfusion injury in the pig retina

**Published:** 2010-11-06

**Authors:** Bodil Gesslein, Gisela Håkansson, Lotta Gustafsson, Per Ekström, Malin Malmsjö

**Affiliations:** Department of Ophthalmology, Lund University, Sweden

## Abstract

**Purpose:**

Numerous studies have been performed aimed at limiting the extent of retinal injury after ischemia, but there is still no effective pharmacological treatment available. The aim of the present study was to examine the role of tumor necrosis factor (TNF)α and its receptors (TNF-R1 and TNF-R2), especially considering the neuroretina and the retinal vasculature since the retinal blood vessels are key organs in circulatory failure.

**Methods:**

Retinal ischemia was induced in pigs by elevating the intraocular pressure to 80 mmHg in one eye, while the other eye served as a control (sham-operated). One hour of ischemia was followed by 5 or 12 h of reperfusion. Retinal circulation was examined in vivo by fundus imaging and fluorescein angiography. TNF-α levels were measured in the vitreous using an angiogenesis antibody array test. The presence and amounts of TNF-α, TNF-R1, and TNF-R2 were investigated in the neuroretina and in the retinal blood vessels, using immunofluorescence staining and real-time PCR techniques.

**Results:**

Fundus imaging showed obstructed blood flow when ischemia was induced, and reperfusion was clearly visualized using fluorescein angiography. Ischemia resulted in elevated levels of TNF-α protein in the vitreous and *TNF-α* mRNA in the neuroretina. TNF-α immunofluorescence staining was localized to the Müller cells and the outer plexiform layer of the neuroretina. The expression of *TNF-R1* and *TNF-R2* mRNA was increased in both the neuroretina and retinal arteries following ischemia-reperfusion. Immunofluorescence double staining for TNF-R1 and either smooth muscle actin or 4',6-diamidino-2-phenylindole (DAPI) indicated expression in the cell membranes of the vascular smooth muscle cells. Double staining with TNF-R1 and calbindin showed localization to the horizontal cells in the outer plexiform layer of the neuroretina.

**Conclusions:**

Retinal ischemia results in increased expression of TNF-α and its receptors (TNF-R1 and TNF-R2). Cellular signaling pathways involving TNF may be important in the development of retinal injury following ischemia and thus an interesting target for future development of pharmacological therapeutics.

## Introduction

Diabetes, vein thrombosis, and arterial occlusion are the most common causes of retinal ischemia and a major cause of sight-threatening complications and blindness [1]. It is important to limit the extent of the ischemic injury, for which there is no successful pharmacological approach at present. If more knowledge is gained of the effects of ischemia on the retina at a cellular and molecular level, pharmacological targets may be revealed.

Retinal ischemia is most probably multifactorial and probably has numerous overlapping signaling pathways that may contribute to the injury. Tumor necrosis factor α (TNF-α) is interesting in the context of retinal circulatory failure in that it is involved in mediating the harmful processes that are initiated following stroke [2-4] and ischemic heart disease [5,6]. Both TNF-α mRNA and protein expression have been shown to be increased in the retina following ischemia [7-10]. TNF-α is an inflammatory cytokine and has been suggested to stimulate angiogenesis following ischemia through the induced expression of many angiogenesis-related genes [11-13]. It is known as a strong immunomediator and pro-inflammatory cytokine, which is rapidly upregulated in the brain after injury [4]. Furthermore, TNF-α is responsible for some of the signaling events within cells that lead to necrosis or apoptosis. It has been identified as a highly cytotoxic cytokine for tumor cells, causing tumor necrosis in vivo and showing cytolytic activity against tumor cells in vitro [14]. TNF-α can be both membrane bound and soluble in tissues and its effects are mediated by the TNF receptors TNF-R1 and TNF-R2 [15]. TNF-R1 can be fully activated by both the membrane-bound and soluble forms of TNF-α, whereas TNF-R2 only responds to the membrane-bound form. The activation of TNF-R1 leads to the activation of multiple apoptotic pathways involving the activation of the pro-death Bcl-2 family of proteins, reactive oxygen species [16], and c-jun NH_2_-terminal kinase [15]. These pathways are closely interlinked and mainly act on mitochondria, resulting in apoptosis. TNF-α/TNF-R1 may simultaneously activate the nuclear factor-κB (NF-κB) pathway, which can inhibit the TNF-α-induced cell-death process [17]. In contrast, TNF-R2 may serve to potentiate the effects of TNF-R1 in promoting cell death or inflammation [15,16]. Both TNF-R1 and TNF-R2 have been shown to be increased in retinal ischemia in mice where TNF-R1 increased neuronal death and TNF-R2 promoted neuroprotection [7]. The function and importance of the TNF-α system in retinal circulatory failure hence appears to be complex.

The aim of the present study was to examine the role of TNF-α and its receptors in retinal ischemia-reperfusion injury in detail, with regard to both the neuroretina and the retinal vasculature, by using the porcine model of retinal ischemia-reperfusion induced by elevating the intraocular pressure (IOP) [18-20]. To achieve this, several methodologies were used, including monitoring of the retinal vasculature in vivo using fundus imaging and fluorescein angiography. Furthermore, TNF-α levels were measured in the vitreous using an angiogenesis antibody array test, whereas the presence and levels of TNF-α, TNF-R1, and TNF-R2 were investigated in both the neuroretina and in the retinal blood vessels, separately, using real-time PCR and immunofluorescence staining techniques.

## Material

### Animals and anesthesia

A total of 15 healthy landrace pigs (10 female and 5 male) with a mean bodyweight of 70 kg were used for this study (conventional pig breeder, Lund, Sweden). Before surgery the animals were fasted overnight with free access to water. An intramuscular injection of 15 mg/kg bodyweight ketamine (100 mg/ml Ketaminol vet^™^; Farmaceutici Gellini S.p.A., Aprilia, Italy) was used in combination with 2 mg/kg bodyweight xylazine (20 mg/ml Rompun vet^™^; Bayer AG, Leverkusen, Germany) for premedication. After induction of anesthesia with thiopental, 12.5 mg/kg bodyweight (Pentothal; Abbott, Stockholm, Sweden), the animals were orally intubated with cuffed endotracheal tubes. Anesthesia was maintained by continuous intravenous infusion of 20 mg/ml propofol (Diprivan^™^; Astra Zeneca, Södertälje, Sweden), at a dosage of 0.1–0.2 mg/kg/min, in combination with intermittent fentanyl administration (Fentanyl B. Braun; B. Braun Malsungen AG, Melsungen, Germany) at approximately 3.5 μg/kg/h. Mechanical ventilation was established with a Siemens-Elema 900B ventilator (Siemens Healthcare Diagnostics, Skärholmen, Sweden) in the volume-controlled mode and adjusted to obtain normocapnia. The animals were ventilated with a mixture of oxygen (70%) and dinitrous oxide (30%). During the procedure, the mean arterial blood pressure of the pigs was 92±7 mmHg. After completion of the experiments, the animals were euthanized by a lethal injection of potassium, 2 mmol/kg (ADDEX potassium chloride; Fresenius KABI SE, Uppsala, Sweden).

### Ethics

All procedures and animal treatment took place in accordance with the guidelines of the Ethics Committee of Lund University, the Institute for Laboratory Animal Research (Guide for the Care and Use of Laboratory Animals), and the Association for Research in Vision and Ophthalmology statement of Use of Animals in Ophthalmic and Vision Research.

### Retinal ischemia-reperfusion

The posterior chamber in one eye was cannulated with a 30-gauge needle, and the IOP was increased to 80 mmHg by continuous infusion of a balanced salt solution for ophthalmic irrigation (Content per ml: 0.48 mg CaCl_2_*2H_2_O, 0.75 mg KCl, 0.30 mg MgCl_2_*6H_2_O, 3.90 mg Na-Acetate*3H_2_O, 1.70 mg Na-Citrate*2H_2_O 6.40 mg NaCl, citric acid to adjust pH and water for injection; Amo^TM^ Endosol^TM^; AMO Groningen BV, Groningen, the Netherlands). The IOP was verified using a Tono-Pen®XL tonometer (Medtronic, Jacksonville, FL). The other eye served as a control, and the same surgical procedure was performed except for elevation of the IOP (this is referred to as the “sham-operated” eye in the text and figures). After 1 h, the cannulation needles were removed, and reperfusion of the retinal vasculature was allowed to take place for 5 or 12 h during anesthesia. Ischemia was confirmed by indirect ophthalmoscopic examination and fundus imaging as described below.

### Fundus imaging and fluorescein angiography

The pupils were dilated with topical cyclopentolate hydrochloride (1%, Cyclogyl; Alcon Laboratories Inc., Fort Worth, TX) to a pupil diameter of 8–10 mm. The IOP was increased gradually from the normal level at10–20 to 40±5, 60±5, 70±5, and 80±5 mmHg, and the fundus was imaged continuously at each pressure level. Reperfusion was monitored using fluorescein angiography. Fluorescein (5 ml, 100 mg/ml, Fluorescite; Alcon) was injected when the IOP was stable at 80 mmHg. The IOP was then allowed to normalize, and the fundus was imaged during pressure normalization. The retinal angiographic images were acquired with a RetCam 3 fundus camera (Clarity Medical Systems Inc., Pleasanton, CA). Standard angiograms were recorded with a blue filter after the injection of fluorescein dye. A wire ophthalmologic speculum was required to separate the pigs’ eyelids for angiography. Video images were recorded at a rate of 30 frames per second, until full arteriovenous filling was observed.

### Tissue preparation

After 5 or 12 h of reperfusion, both eyes of each pig were enucleated under anesthesia (including the optic nerve). The eyes were dissected, the anterior segment was removed, and the vitreous humor collected. The vitreous was homogenized by aspiration through a Pasteur pipette and then centrifuged for 30 min at 16,000× g at 4 °C to precipitate insoluble tissue. The vitreous homogenate was snap frozen in liquid nitrogen and stored at −80 °C until further analysis.

One limitation of the present study was that the retinal arteries and blood vessels of the eyes exposed to 5 h and 12 h of ischemia-reperfusion were dissected in two different ways and that the retina used for real-time PCR (see below) did not consist of whole retinas but pieces. However, since both eyes from a particular pig were dissected in the same way, it is unlikely that the dissection technique would have affected the results and conclusions drawn from the comparison of the ischemia-reperfused eye and the control eye from each animal. Dissection was performed as follows: A slice along the superior–inferior axis containing the optic nerve head of the eye was first removed. This was later prepared for immunofluorescence and histological investigation (see below). The retina was then dissected free from the sclera and retinal pigment epithelium in the remainder of the eye. Regarding the eyes exposed to 5 h of ischemia-reperfusion, the large retinal arteries were isolated from the neuroretina by careful dissection in a balanced salt solution for ophthalmic irrigation at 4 °C. Pieces of each neuroretina and the retinal arteries were frozen separately. Regarding the eyes exposed to 12 h of ischemia-reperfusion, the neuroretina was isolated by dissecting pieces lacking major blood vessels, in the same balanced salt solution, at 4 °C and frozen as above. After removing pieces of neuroretina, the rest of the retina was placed in distilled water on ice for 1 h. During this time, the tissue was gently and repeatedly aspirated through a Pasteur pipette under magnification, taking care not to damage the blood vessels. The blood vessels were collected and then frozen. The neuroretina and retinal arteries or blood vessels were collected, frozen, and stored at −80 °C until used for real-time PCR experiments.

### Histology

Slices from both eyes were fixed in 4% paraformaldehyde for 4 h. After fixation the tissue was rinsed in 0.1 M Sørensen’s phosphate buffer (28 mM NaH_2_PO_4_ and 72 mM Na_2_HPO_4_; pH 7.4; Sigma-Aldrich, St. Louis, MO) and then washed in the same solution with increasing concentrations of sucrose (10%–25%; Sigma-Aldrich). The specimens were embedded in 30% egg albumin and 3% gelatin (Sigma-Aldrich) and were stored at −80 °C until sectioning. The specimens were sectioned at 12 µm in a cryostat (Microm HM500M; Thermo Scientific, Walldorf, Germany) and placed on microscope slides (Menzel, Braunschweig, Germany), three sections on each slide. The slides were allowed to dry at room temperature for 30–60 min and were then stored at −20 °C until used.

### Fluorescent immunohistochemistry

The slides were dried at room temperature for 15 min and permeabilized in PBS (0.14 M NaCI, 0.01 M PO_4_ buffer, 0.003 M KCI, pH 7.45) plus 0.25% Triton X-100 (2×5 min). Blocking solution, containing PBS+0.25% Triton X-100+1% BSA (BSA) and an additional 5% normal serum was added to the slides to avoid nonspecific binding of antibodies. The slides were incubated for 1 h at room temperature. Specimens were incubated overnight at 4 °C with the same blocking solution but with 2% normal serum instead and the primary antibody of interest: 1:400 rabbit polyclonal TNF-R1 antibody (ab19139; Abcam Biotechnology, Cambridge, UK), 1:400 goat polyclonal TNF-α (M-18) antibody (sc-1348; Santa Cruz Biotechnology, Heidelberg, Germany), 1:200 mouse monoclonal calbindin antibody (C9848; Sigma-Aldrich, St. Louis, MO), and/or 1:200 mouse monoclonal anti-smooth muscle actin (sc-53015; Santa Cruz Biotechnology).

The slides were rinsed in PBS (3×15 min), incubated in blocking solution with the appropriate secondary antibody—1:100 fluorescein isothiocyanate (FITC) swine antirabbit (DakoCytomation, Glostrup, Denmark), 1:400 Alexa 488 donkey antigoat (Invitrogen, Carlsbad, CA), 1:300 Texas red donkey antimouse (Jackson ImmunoResearch, West Grove, PA)—and incubated at room temperature for 1 h. The slides were then washed in PBS (3×15 min) and mounted in antifading mounting medium with or without 4´,6-diamidino-2-phenylindole (DAPI; Vectashield; Vector Laboratories Inc., Burlingame, CA). Slides destined for use with the primary antibody against TNF-α were placed in citric acid (pH 6.0) in a microwave oven at full effect for 7 min before the blocking step to open up the epitopes. Sections incubated without primary or secondary antibody were used as negative controls to verify the lack of autofluorescence and nonspecific secondary antibody staining. The staining location was analyzed using a light microscope equipped for fluorescence microscopy (Olympus BX60; Olympus, Tokyo, Japan). Photographs were taken with a digital camera (Pixera Pro 600ES; Pixera, Los Gatos, CA) mounted on the microscope.

### RNA extraction

An Allprep DNA/RNA/Protein Mini Kit (Qiagen, Valencia, CA) was used for the extraction of RNA. Briefly, the tissue was homogenized in buffer RLT, including β-mercaptoethanol, using a stainless steel bead and a Qiagen TissueLyser. The stainless steel bead was removed and the samples were centrifuged at 8,000× g. The supernatant was then transferred to an Allprep DNA spin column and centrifuged at 8,000× g. Ethanol (99.5%) was added to the flow through, and the samples were transferred to an RNeasy (Qiagen) spin column and centrifuged. On-column DNase digestion was performed according to the manufacturer’s instructions (RNase-Free DNase Set, 50; Qiagen). The column was washed with RW1 buffer, and RPE (Qiagen) buffer, and the RNA was eluted in RNase-free water: 50 μl for neuroretina specimens and 30 µl for the retinal arteries/blood vessels. Following extraction, the RNA concentration and purity was measured by the absorbance at 260 nm (A_260_/A_280_ ratio) using a spectrophotometer (TECAN, Männedorf, Switzerland). All samples were stored at -80 °C until further analysis.

### cDNA transcription and real-time PCR

Reverse transcription of total RNA to cDNA was performed using the Taqman GeneAmp RNA PCR kit (N808–0234; Applied Biosystems, Foster City, CA) in a Perkin-Elmer DNA Thermal Cycler (Applied Biosystems). cDNA was synthesized from 1 µg total RNA in a 50-µl reaction with a master mix containing a reverse transcriptase and random hexamers, which act as primers. The reaction was run at 25 °C for 10 min, 37 °C for 60 min, and 95 °C for 5 min. Following reverse transcription, 50 µl double distilled H_2_O was added to neuroretina specimens to a final volume of 100 µl, and all the cDNA samples were stored at -80 °C. Real-time PCR was performed in a GeneAmp 7300 Real Time PCR system, using the Gene Amp Power SYBR^®^ Green kit (Applied Biosystems). The cDNA obtained as described above was used as a template in a 25-µl reaction. A control without template was included in all experiments. Forward and reverse primers were designed using the Primer3 Input, version 0.4.0 [21] (Table 1). The results were calculated relative to the amount of the housekeeping genes β-actin (*Actb*) and elongation factor-1α (*EF-1α*) since these are believed to be continuously expressed at constant amounts in cells [22]. The primer sequences for these housekeeping genes are given in Table 1.

**Table 1:** Real-time PCR primers. Gene name, GenBank number and primer sequence for primers used in real-time PCR experiments.

**Gene name**	**GenBank number**	**Forward primer sequence**	**Reverse primer sequence**
*TNF-α*	EU682384	CCACCAACGTTTTCCTCACT	TAGTCGGGCAGGTTGATCTC
*TNF-R1*	NM_213969	GCCACAAAGGCACCTACCTA	GACATTTCACTCCGGCACTT
*TNF-R2*	EU116354	CACCAGGCTTCCGAATACAA	ACGCAGAAACCGAGTTCCA
*EF-1α*	AM040195	GCTGACTGTGCTGTCCTGATT	TGTAGGCCAGAAGAGCATGCT
*Actb*	U07786	CCTTCAACTCGATCATGAAGTGC	CGTAGAGGTCCTTCCTGATGTCC

Gene name, GenBank number and primer sequence for primers used in real-time PCR experiments.

The forward and reverse primers were dissolved in RNase free water and a mixture of forward and reverse primers was made. A master mix containing RNase-free water, Power SYBR^®^ Green (Applied Biosystems) and the chosen forward and reverse primers (at a final concentration of 200 nM) was added to the wells. One microliter cDNA from the specimens containing neuroretina (sham-operated or ischemia-reperfusion eyes) or retinal arteries/blood vessels (sham-operated or ischemia-reperfusion eyes) was added to the wells. The real-time PCR reaction was performed with the following profile: 1 cycle of 50 °C for 2 min and 95 °C for 10 min, followed by 40 cycles of 95 °C for 15 s and 60 °C for 1 min. This was followed by a dissociation step: 95 °C for 15 s, 60 °C for 30 s, and 95 °C for 15 s. One standard curve was obtained for each pair of primers to confirm that the primers were optimal and that the cDNA had been amplified with the same efficiency during the real-time PCR process. The cycle threshold (C_T_) can be expressed in terms of the amplification efficiency (E), and the cDNA concentration:

$${\text{C}}_{\text{T}}={\left[\text{log }\left(\text{1}+\text{E}\right)\right]}^{-\text{1}}\text{log }\left(\text{concentration}\right),$$

where the amplification efficiency has the optimal value of 1. The amount of *TNF-α*, *TNF-R1*, and *TNF-R2* mRNA in the specimens was calculated relative to the amount of *β-actin* and *EF-1α* mRNA in the same sample using the relation:

$${\text{X}}_{0}/{\text{R}}_{0}={\text{2C}}_{\text{TR}}-{\text{C}}_{\text{TX}},$$

where X_0_=original amount of *TNF-α*, *TNF-R1*, or *TNF-R2* mRNA; R_0_=original amount of *Actb* mRNA; C_TR_ is the C_T_ value for *β-actin*; and C_TX_ is the C_T_ value for *TNF-α*, *TNF-R1*, or *TNF-R2*.

### Angiogenesis antibody array

TNF-α protein levels were determined in vitreous extracts from sham-operated and ischemia-reperfusion eyes following 12 h of reperfusion (n=6) using angiogenesis antibody array membranes (MA6310; Panomics, Fremont, CA). The assay was performed according to the according to the manufacturer’s instructions. In brief, the array membranes were blocked and rinsed before incubation with vitreous extracts for 1 h at room temperature. Membranes were subsequently rinsed and incubated with Biotin-Conjugated Anti-Cytokine/Anti-Angiogenesis Mix and then developed with Streptavidin-HRP Conjugate (both supplied in the kit). Protein spots on membranes were visualized using a Fujifilm LAS-1000 Luminescent Image Analyzer (Fujifilm, Stamford, CT). The spot intensity was quantified using Image J software [23].

### Calculations and statistics

Statistical analysis was performed using the paired Student ratio *t* test with the Bonferroni correction for multiple comparisons. Calculations and statistics were performed using GraphPad Prism 5.0 software (GraphPad Software, Inc., La Jolla, CA). Differences were considered significant when p<0.05. Results are presented as means±the standard error of the mean.

### Limitations

The pigs in this study were anesthetized throughout the study, and the interaction between ischemia and anesthesia is not known but may have impacted the results. To limit the impact on the results and conclusions drawn from the study, the results from the ischemia-reperfusion eye were always compared with the results from the sham-operated eye in the same animal. The other option would be to wake the animals and re-anesthetize them again after the reperfusion; this would, however, subject the animals to increased stress, which is also known to induce metabolic changes. Taken together, we believe that the conclusions drawn from the study have not been affected.

A limitation to the present study is that satisfactory results could not be achieved for immunofluorescence with TNF-R2. Our real-time PCR shows an effect of ischemia-reperfusion on TNF-R2, and TNF-R2 has previously been suggested to be neuroprotective [7]. This is a consequence of working with the pig as an animal model since very few antibodies are produced against pig proteins and none of the tested human/rat/mouse TNF-R2 antibodies gave reliable and clear-cut results in our pig model.

## Results

### Fundus imaging

**Figure 1:**
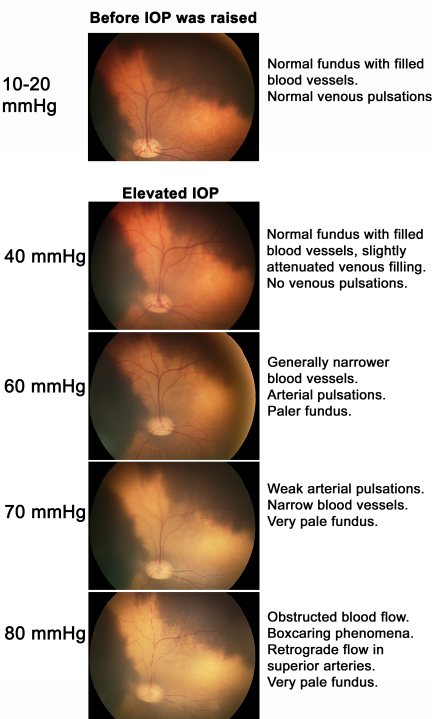
Fundus images from an eye subjected to successively increasing intraocular pressure. At normal pressure (10–20 mmHg) the pigs had a normal fundus with filled blood vessels. The filling of the blood vessels decreased and the fundus became paler as the intraocular pressure (IOP) increased. At an IOP of 80 mmHg, blood flow was completely inhibited. IOP is given with ±5 mmHg variability.

The retinal blood vessels were visualized by fundus imaging before the IOP was elevated and at increasing levels of IOP. Before the IOP was elevated (normal pressure of 10–20 mmHg), the pigs had a normal fundus with filled blood vessels and venous pulsations were seen. When the pressure was increased to 40 mmHg, the veins were slightly less filled and there were no venous pulsations. At 60 mmHg, blood perfusion was still maintained via both arteries and veins, although these blood vessels appeared narrower. Arterial pulsations could be observed, and the fundus was paler. At a pressure of 70 mmHg, the blood vessels were thin and the blood flow was slow and limited, with weak arterial pulsations. At this pressure, the fundus was clearly pale. At the highest IOP, 80 mmHg, blood flow was completely obstructed. Boxcaring (break up of blood flow into clumps of red blood cells giving an appearance of boxcars in the vessels) phenomena were seen in both veins and arteries, and a retrograde flow was seen in superior arteries. Figure 1 shows representative examples of fundus images at these different pressure levels.

### Fluorescein angiography

**Figure 2:**
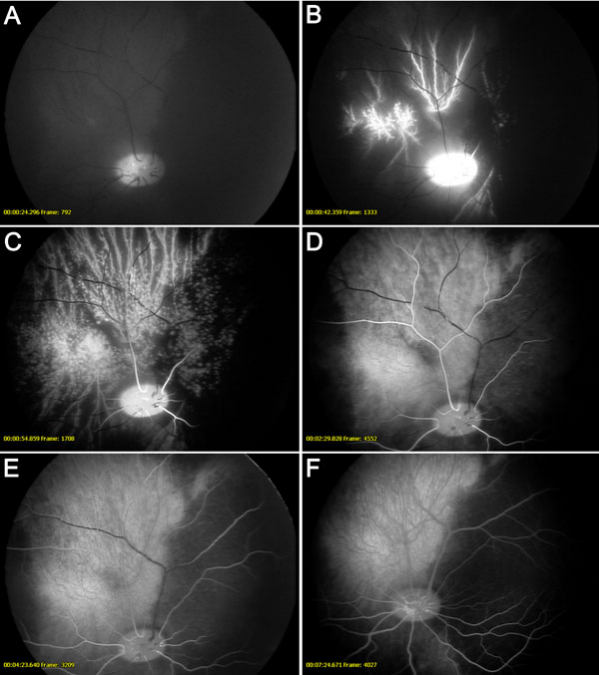
Fundus images during fluorescein angiography of a retina that is exposed to high intraocular pressure (80 mmHg). **A**: At 80 mmHg blood flow was completely obstructed. The intraocular pressure (IOP) was then gradually reduced and the following events were observed: (**B**) hyperfluorescence of the optic nerve head and filling of the large blood vessel of the choroidal circulation; (**C**) distinct pulsations and slight filling of the retinal arteries (at this stage, medium-sized choroidal vessels, but not choriocapillaries, were perfused); (**D**) blood flow through the arteries was then restored, but still no venous filling; (**E**) micro-emboli were seen to move slowly out of the veins to allow reperfusion; (**F**) and finally, background fluorescence after angiography.

Fluorescein angiography was performed at the highest IOP (80 mmHg) when no perfusion of blood vessels could be seen. The IOP was then gradually reduced and the following events were observed. First, there was hyperfluorescence in the optic nerve head and filling of the large blood vessel of the choroidal circulation. This was followed by distinct pulsations and slight filling of the retinal arteries. At this stage medium-sized choroidal vessels, but not choriocapillaries, were perfused. Blood flow through the arteries was then restored, but there was still no venous filling. In the next stage, micro-emboli were seen to move slowly out of the veins to allow reperfusion. See Figure 2 for fluorescein angiography images.

### Real-time PCR control experiments

Similar patterns of *TNF-α*, *TNF-R1*, and *TNF-R2* mRNA expression in the real-time PCR experiments were seen in the neuroretina for pigs subjected to 12 h of ischemia-reperfusion when using *Actb* and *EF-1α* as reference genes. Examples of comparisons of *Actb* and *EF-1α* as reference gene are as follows:

*TNF-α* was 1.72±0.93 with *Actb* and 2.29±0.44 with *EF-1α* as reference gene (p>0.3). *TNF-R1* was 2.47±0.20 with *Actb* and 2.34±0.29 with *EF-1α* as reference gene (p>0.3). *TNF-R2* was 1.19±0.20 with *Actb* and 1.07±0.14 with *EF-1α* as reference gene (p=0.2171). The results suggest that both *Actb* and *EF-1α* were trustworthy as references genes and from here on the results in figures and text are given in relation to *Actb*. The standard curves for each primer pair had similar slopes: 3.3 for *TNF-α*, 3.4 for *TNF-R1*, 3.1 for *TNF-R2,* and 3.3 for *Actb*, indicating that they were amplified with similar efficiency.

### Tumor necrosis factor α after retinal ischemia-reperfusion

**Figure 3:**
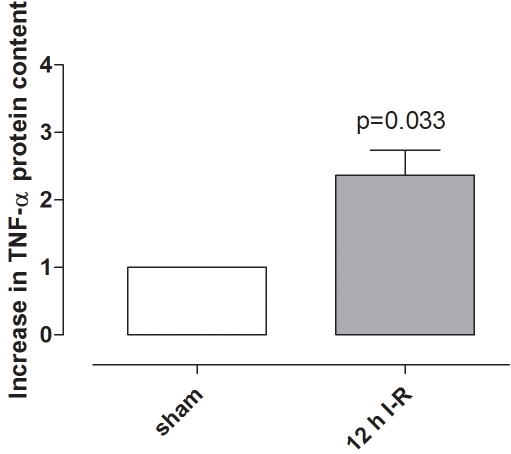
Tumor necrosis factor α in the vitreous. Extracts of vitreous were incubated with an angiogenesis antibody array membrane and tumor necrocsis factor α (TNF-α) protein levels in the vitreous in eyes exposed to 12 h of ischemia-reperfusion (n=6), were compared to sham-operated eyes (n=6). The graph shows the mean values±standard error of the mean obtained from quantification of the spot intensity and is expressed as the number of times by which the TNF-α increased in ischemia-reperfusion compared with the sham-operated eyes. Statistical analysis was performed using paired Student ratio *t* test. Note that TNF-α protein levels in the vitreous humor are higher following ischemia-reperfusion.

**Figure 4:**
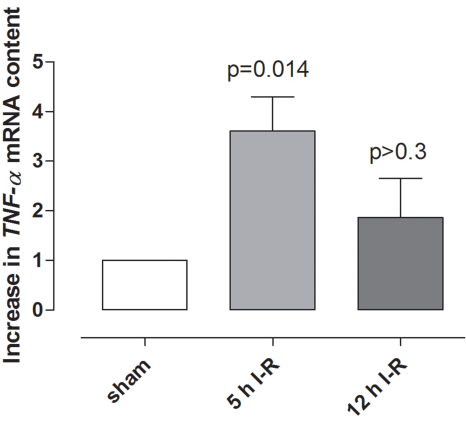
Tumor necrosis factor α *(TNFα)* mRNA expression in the neuroretina. *TNF-α* mRNA expression levels in the neuroretina of sham-operated eyes were compared to eyes subjected to ischemia followed by 5 h (n=4) and 12 h (n=8) of reperfusion (I-R), assessed by real-time PCR. The mRNA levels were calculated relative to the housekeeping gene *Actb*. Results are expressed as the number of times by which the *TNF-α* mRNA increased in the neuroretina from ischemia-reperfusion eyes compared with the sham-operated eyes (mean±SEM). Statistical analysis was performed using paired Student ratio *t* test with the Bonferroni correction.

**Figure 5:**
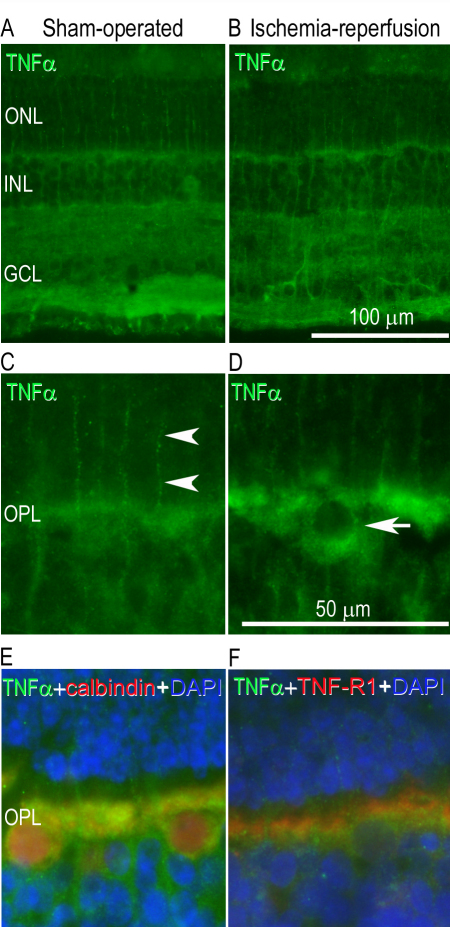
Tumor necrosis factor α (TNF-α) immunofluorescence in the neuroretina. TNF-α immunofluorescence staining was detected in the outer plexiform layer and in the Müller cells of the neuroretina in (**A**) a sham-operated eye and (**B**) the fellow eye subjected to ischemia and 12 h of reperfusion. **C**-**D**: Enlargement showing TNF-α staining in the (**C**) Müller cell processes (arrow heads) and (**D**) cellbodies of the outer plexiform layer (arrow). **E**: Enlargement showing double staining with TNF-α and calbindin (a horizontal cell marker) and (**F**) TNF-α with its receptor TNF receptor 1 (TNF-R1). DAPI was used to label the cell nuclei. The different images are from separate sections of the retina. Abbreviations used in the figure are outer nuclear layer (ONL), inner nuclear layer (INL), ganglion cell layer (GCL), and outer plexiform layer (OPL).

TNF-α protein levels in the vitreous were higher in ischemia-reperfusion eyes than in sham-operated eyes (Figure 3). *TNF-α* mRNA levels were elevated in the neuroretina following ischemia-reperfusion for 5 h but were not significantly altered after 12 h of reperfusion (Figure 4). TNF-α immunofluorescence staining was localized to the Müller cells, as judged by staining of the radial processes, and in the outer plexiform layer (OPL) of the neuroretina (Figure 5).

### Tumor necrosis factor receptor 1 and tumor necrosis factor receptor 2 after retinal ischemia-reperfusion

**Figure 6:**
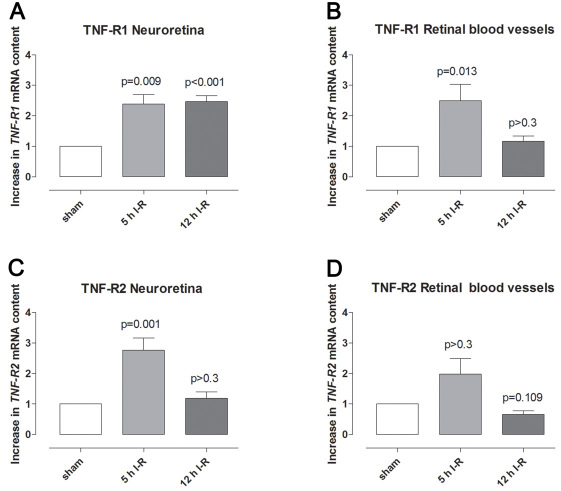
Tumor necrosis factor (*TNF*)-*R1* and *TNF-R2* mRNA expression. **A**-**B**: *TNF-R1* and (C-**D**) *TNFR2* mRNA expression levels in (**A**, **C**) the neuroretina and (**B**, **D**) retinal blood vessels from sham-operated eyes were compared to eyes subjected to ischemia followed by 5 h (n=7) and 12 h (n=8) of reperfusion (I-R), assessed by real-time PCR. The mRNA levels were calculated relative to the housekeeping gene *Actb*. Results are expressed as the number of times by which the *TNFR1* and *TNF-R2* mRNA increased in the neuroretina from ischemia-reperfusion eyes compared with the sham-operated eyes (mean±SEM). Statistical analysis was performed using paired Student ratio *t*-test with the Bonferroni correction.

**Figure 7:**
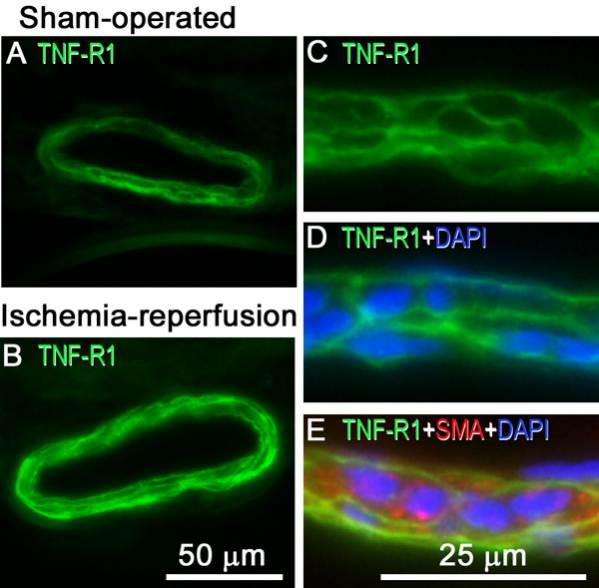
Retinal artery tumor necrosis factor (TNF)-R1 immunofluorescence. Images show representative examples of TNF-R1 immunofluorescence staining in an artery from (**A**) a sham-operated eye and (**B**) the fellow eye subjected to ischemia and 12 h of reperfusion. **C**-**E**: Enlargements show double staining for (**C**-**E**) TNF-R1 and (**E**) the smooth muscle cell marker, smooth muscle actin (SMA). It can be shown that TNF-R1 and SMA co-localize in the smooth muscle cells in the blood vessels. **D**-**E**: Furthermore, staining with DAPI (which labels the cell nuclei) showed that TNF-R1 was located in the cell membrane of the smooth muscle cells. The different images are from separate sections.

**Figure 8:**
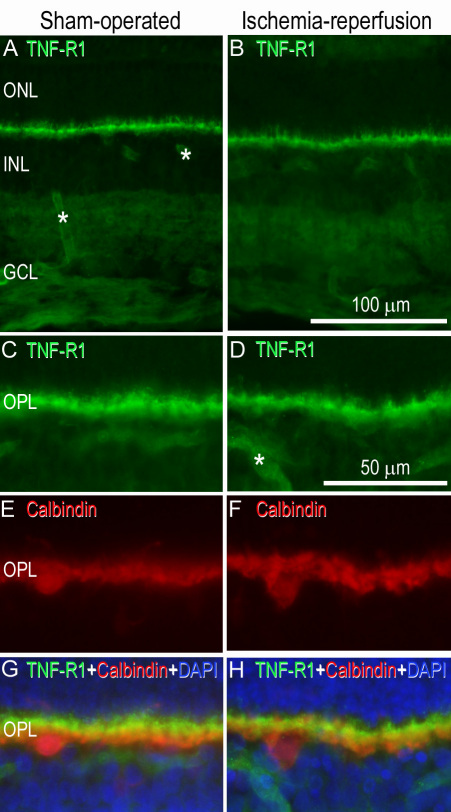
Tumor necrosis factor (TNF)-R1 immunofluorescence staining in the neuroretina. Representative examples of (**A**) a sham-operated eye and (**B**) the fellow eye subjected to ischemia and 12 h of reperfusion showing TNF-R1 staining in the outer plexiform layer of the neuroretina. **C**-**H**: The smaller pictures are enlargements showing double staining for (**C**-**D**) TNF-R1 and (**E**-**F**) calbindin antibodies. Co-localization (**G**-**H**) could be seen in the horizontal cells. **G**-**H**: DAPI showed staining of the nucleus. Asterisks indicate labeled blood vessels. The original and enlarged images are from separate sections. Abbreviations used in the figure are outer nuclear layer (ONL), inner nuclear layer (INL), ganglion cell layer (GCL) and outer plexiform layer (OPL).

*TNF-R1* and *TNF-R2* mRNA levels were generally higher in the neuroretina and retinal blood vessels after ischemia-reperfusion than in sham-operated eyes (for detailed results see Figure 6). TNF-R1 immunofluorescence staining was primarily located in the cell membranes of the smooth muscle cell layer of the blood vessels and was confirmed by triple staining for TNF-R1, smooth muscle actin, and 4',6-diamidino-2-phenylindole (DAPI; which labels the cell nuclei; Figure 7). Since TNF-R1 was primarily visualized in the smooth muscle cell layer, it was more abundant in arteries than veins. In the neuroretina, TNF-R1 staining was primarily observed in the OPL. Double staining with TNF-R1 and calbindin, a marker for horizontal cells [24], suggested TNF-R1 expression in the latter cell type (Figure 8). Double staining with TNF- α and TNF-R1 further showed co-localization of the cytokine and its receptor in the OPL.

## Discussion

In the present study, the retinal vasculature was monitored in vivo using fundus imaging and fluorescein angiography after elevating the IOP to induce ischemia and lowering of the IOP to allow reperfusion. Fundus imaging showed that the retinal blood vessels were thinner than in normal eyes at an IOP of 60 mmHg, and at 80 mmHg there was no visible blood flow through the retinal blood vessels. Fluorescein angiography confirmed that there was no perfusion of retinal blood vessels at an IOP of 80 mmHg, which is a pressure used in this and other studies to create retinal ischemia [18-20]. La Cour et al. [25] showed that the optic nerve oxygen tension remained relatively constant for an IOP up to 40 cm H_2_O (29 mmHg), but after the IOP was increased to 80 cm H_2_O (59 mmHg), the optic nerve oxygen tension fell sharply, and further increases in IOP above 120 cm H_2_O (88 mmHg) did not influence the oxygen tension of the optic nerve. This clearly indicates that a pressure of 80 mmHg, which has been used in this and previous studies [18-20], is sufficient to hinder blood perfusion of the retina. It furthermore fits well with other studies that have verified that elevating IOP to 80 mmHg results in ischemic injury to the retina, as shown by increased number of terminal deoxynucleotidyl transferase dUTP nick end labeling (TUNEL) positive cells, pyknotic cell nuclei, glial fibrillary acidic protein activation [19], and multifocal electroretinogram changes [20]. However, the retina is relatively resistant to an ischemic insult compared to the brain, which suffers widespread injury after only a few minutes of cerebral ischemia. The relative resistance of the retina to ischemia can be explained by local energy substrates that are present in the vitreous and the retina and the ability to extract ATP (ATP) in the absence of oxygen; in addition the edematous retina does not obstruct the microvasculature as the thin retinal tissue has the vitreous cavity to expand into (in contrast to the cranium that limits the brain) [1].

In the present study, retinal reperfusion following ischemia was visualized with fluorescein angiography. There is increasing evidence that reperfusion generates detrimental intracellular signaling cascades and contributes to enlargement of the ischemic injury [1], and such reperfusion injury may be the result of reactive hyperemia following the period of ischemia. For example, transient hyperemia, with an increase in blood flow to almost twice the baseline (192%), has been demonstrated in cerebral ischemia in rabbits [26]. Similarly, in a cat model of ischemia-reperfusion employing a similar approach to that used here, retinal and choroidal blood flows were nearly abolished during the ischemic insult and the retinal blood flow was about twice the baseline level after only 5 min of reperfusion [27]. It is believed that retinal ischemia in combination with the ensuing reperfusion triggers intracellular signaling cascades that lead to retinal injury [1].

TNF-α is one of many factors that may contribute to the development of ischemic injury. TNF-α is known to affect the growth, differentiation, survival, and physiologic function of a variety of cells, including those in the immune system, astrocytes, microglia, and smooth muscle cells [15]. In the present study, TNF-α protein levels were elevated in the vitreous following ischemia-reperfusion. The reason for this cannot be deduced from the present study but may be related to the increased expression of TNF-α in the neuroretina. TNF-α has previously been shown to be increased in blood serum from mice subjected to ischemia-reperfusion [9] and in vitreous [28] and blood serum [29] from patients with proliferative diabetic retinopathy. The present results also show that the *TNF-α* mRNA level in the neuroretina was significantly increased after ischemia and 5 h reperfusion, while after 12 h of reperfusion no significant increase was observed when compared to the sham-operated eyes. Similar results have been seen in a mouse model of central retinal artery occlusion, where *TNF-α* mRNA levels were increased early but then almost reached control levels again after 7 days [9]. Furthermore, an early increase in *TNF-α* mRNA has also been seen in a rat model of pressure-induced ischemia-reperfusion [8]. These results indicate that TNF-α is expressed early after retinal ischemia-reperfusion injury and then returns to baseline levels again. In the present study TNF-α immunofluorescence was detected in the OPL and in glial processes corresponding to Müller cells. This is in line with a study from Fontaine et al. where staining of Müller processes was shown in the neuroretina following ischemia-reperfusion in mice [7].

TNF-α has been suggested to be an inducer of apoptotic cell death via TNF-R1 occupancy in a caspase-mediated pathway, which includes the activation of caspase-8 [30]. The binding of TNF-α to TNF-R1 triggers a series of intracellular events, resulting in the activation of two major transcription factors, NF-κB and c-jun [31]. In a previous study by us using the same model of retinal ischemia-reperfusion, c-jun mRNA and protein levels were shown to be increased in the neuroretina [19]. Taken together, these results suggest that TNF-α is increased in the vitreous and neuroretina during ischemia-reperfusion and thus becomes involved in a signaling cascade that may be detrimental to the retina.

In the present study, the expression of *TNF-R1* and *TNF-R2* mRNA was increased in both the neuroretina and retinal arteries following ischemia-reperfusion. Interestingly, *TNF-R1* and *TNF-R2* mRNA levels have previously been shown to be increased in the brain following ischemia [32,33], and *TNF-R1* mRNA levels have been shown to be increased in the retina in patients with glaucoma [10]. Such observations suggest that TNF-α and its receptors may be involved in causing ischemic damage in nervous tissue, which could occur through a variety of pro-inflammatory effects, such as the activation of microglia and astrocytes, and induction of intercellular adhesion molecule-1 [33,34]. Delayed expression of intercellular adhesion molecule-1 in astrocytes may be regulated by TNF-α and its receptors after brain ischemia [35]. TNF-R1 immunofluorescence was observed in the OPL, which is in accordance with previous studies [7], and showed co-localization with its activator TNF-α.

Inhibitors of TNF-α are now being constructed and include a soluble TNF-α receptor antagonist (etanercept), three anti-TNF-α antibodies (adalimumab, infliximab, and golimumab), and a humanized Fab fragment combined with polyethylene glycol (certolizumab) [36,37]. In the clinic, TNF-α inhibitors are being used with success in the treatment for rheumatoid arthritis, psoriatic arthritis, and Crohn’s disease [36]. In light of this, modulating TNF expression in the retina may be an interesting alternative for the treatment of retinal injury following ischemia. In fact, TNF-α modulation has recently started to be considered in the context of refractory diabetic macular edema or choroidal neovascularization secondary to age-related macular degeneration [38,39]. To further evaluate the importance of TNF in retinal ischemia, the next step is to apply an inhibitor of TNF to ischemic eyes and perform long-term studies to follow the development of injury and to evaluate the effect of blocking the TNF pathway.

In conclusion, ischemia was produced by increasing the IOP to 80 mmHg. At this pressure blood flow to the retina was obstructed, as shown by fundus imaging. After 1 h of ischemia, the IOP was allowed to return to normal levels and the retinal vasculature was reperfused, as shown by fluorescein angiography. Retinal ischemia-reperfusion resulted in elevated levels of TNF-α protein in the vitreous and *TNF-α* mRNA in the neuroretina; the expression of *TNF-R1* and *TNF-R2* mRNA was also increased in both the neuroretina and retinal arteries. These results indicate that the TNF signaling pathways may be interesting targets for the development of pharmacological therapeutics aimed at preventing the development of retinal injury following ischemia.
